# Autografting of bone marrow mesenchymal stem cells alleviates streptozotocin-induced diabetes in miniature pigs: Real-time tracing with MRI *in vivo*

**DOI:** 10.3892/ijmm.2014.1729

**Published:** 2014-04-07

**Authors:** KUANXIAO TANG, XIAOYAN XIAO, DAYUE LIU, YUNFENG SHEN, YINGMING CHEN, YU WANG, BAOYING LI, FEI YU, DEDONG MA, JINHUA YAN, HUA LIANG, DAIZHI YANG, JIANPING WENG

**Affiliations:** 1 Department of Endocrinology, The Third Affiliated Hospital of Sun Yat-sen University, Guangzhou, Guangdong 510630, P.R. China; 2 Department of Geriatrics, Qilu Hospital of Shandong University, Jinan, Shandong 250012, P.R. China; 3 Department of Nephrology, Qilu Hospital of Shandong University, Jinan, Shandong 250012, P.R. China; 4 Department of Vascular Surgery and Radiology, The First Affiliated Hospital of Sun Yat-sen University, Guangzhou, Guangdong 510080, P.R. China

**Keywords:** bone marrow mesenchymal stem cells, autologous transplantation, cell therapeutics, diabetes mellitus type 1, miniature pigs

## Abstract

Cellular replacement therapy for diabetes mellitus (DM) has received much attention. In this study, we investigated the effect of transplantation of autologous bone marrow-derived mesenchymal stem cells (ABMSCs) in streptozotocin (STZ)-induced diabetic miniature pigs. Miniature pig BMSCs were cultured, labeled with superparamagnetic iron oxide (SPIO) and transplanted into the pancreas of diabetic miniature pigs through targeted intervention. Blood glucose levels, intravenous and oral glucose tolerance test (OGTT), serum insulin, C-peptide and islets histology were analyzed. These transplanted cells were then identified by magnetic resonance imaging (MRI). The results showed that transplantation of ABMSCs reversed STZ-induced diabetes in miniature pigs. Blood glucose levels, intravenous, OGTT, serum insulin and C-peptide were significantly recovered in the diabetic minipigs with the autologous BMSC (DMAB) transplantation group. In addition, the number of islets was significantly increased in this group compared to the diabetic minipig control (DMC) group with conventional therapy. These data suggested the implantation of autologous BMSCs for type 1 diabetes treatment can partially restore the function of islet β cells and maintain blood glucose homeostasis. Transplanted autologous BMSCs may improve islet repairing by differentiating for new islets and change pancreatic microcirculation and be identified in a real-time manner using MRI *in vivo*.

## Introduction

Type 1 diabetes mellitus (T1D) is a chronic condition in which the pancreas produces little or no insulin due to autoimmune destruction of insulin-producing β cells in the islets. As diabetes is caused by the loss of a single cell type, cellular replacement therapy for diabetes mellitus (DM), especially T1D, has received much attention. More recently, additional efforts have been focused on the use of both autologous and allogeneic stem cells as sources of new islets (1–5).

Bone marrow-derived mesenchymal stem cells (BMSCs) exhibit considerable developmental plasticity that can be induced to differentiate into insulin-producing cells *in vitro*. Although it is possible to induce β-cell-specific gene expression in murine and human BMSCs *in vitro*, insulin secretion of these differentiated cells is extremely low (2). Findings of previous studies have demonstrated that rodent BMSCs can adopt insulin-expressing phenotype (6), drive the phenotype of human BMSCs by the forced expression of β-cell transcription factors, and generate cells capable of glucose responsive insulin secretion (7). BMSCs transplanted into diabetic mice contribute to endocrine pancreatic regeneration (7–9). BMSCs reversed experimental diabetes *in vivo* by enhancing the regeneration and survival of endogenous β cells rather than repopulating the islets with transdifferentiated β cells (10,11).

The potential mechanisms of these beneficial animal and clinical results from stem cell transplantation remain to be determined. However, the final destination of the cells and their fate following their transplantation *in vivo* has not been clearly determined. In this study, we used streptozotocin (STZ) to induce diabetes in miniature pigs, transplanted autologous BMSCs (ABMSCs) into the pancreas through targeted intervention, identified them *in vivo* by MRI scanning and monitored glycemic status and pancreatic islet β-cell function.

## Materials and methods

### Experimental animals

Tibetan miniature male pigs (3 months old) were purchased from the Guangdong Experimental Animal Center (Guangzhou, China). The animals were housed in a controlled temperature of 22–25°C, with a humidity of 50–70%, as well as air change and lighting, The animals were fed twice daily according to the Larsen protocol revised for the requirements of the present study (commercial miniature pigs mixed fodder 30 g/kg body weight) (14), and allowed access to water *ad libitum*.

### Central venous catheter implantation

A dual lumen venous catheter (5F; Arrow-Howes™ Multi-Lumen Catheter; Teleflex, Inc., Limerick, PA, USA) and ear vein indwelling needle (24G; BD Biosciences, Franklin Lakes, NJ, USA) were surgically inserted into the internal jugular vein and ear vein under general anesthesia. All the procedures were performed in accordance with the principles of Laboratory Animal Care (National Institutes of Health publication no. 85–23, revised 1985) and the animal protocol was approved by the Sun Yat-sen University, Institutional Animal Care and Use Committee (IACUC, Approval ID no.: 2008020102).

### T1D model

The T1D model was induced by intravenous administration of STZ (Sigma-Aldrich, St. Louis, MO, USA) with sodium citrate as the buffer through indwelling catheters, at a dose of 120 mg/kg body weight after 24-h fasting in conscious animals, while the normal control miniature pigs received an injection of the same amount of citric buffer (30). Seventy two hours after the injection of STZ, the fasting blood glucose (FBG) was detected in the subsequent days. Diabetes was confirmed following three consecutive readings of blood glucose ≥16.7 mmol/l (300 mg/dl).

### Animal grouping and treatment

**Figure 1:**
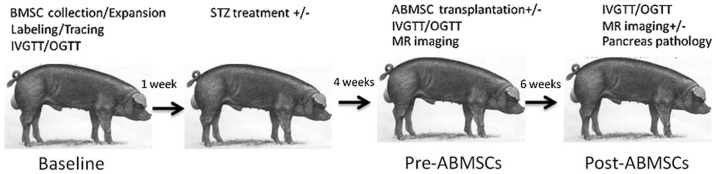
The experimental procedure.

Experimental animals were randomly divided into three groups with 5 miniature pigs in each group: normal controls (NC) group without DM, diabetic minipig control group (DMC) and diabetic minipigs with autologous BMSC transplantation group (DMAB). DMC and DMAB animals received subcutaneous injection of Humulin N (Eli Lilly and Company, Indianapolis, IN, USA) after T1D was confirmed in order to maintain the blood glucose levels of animals at a the range of 5.0–10.0 mmol/l. After 4 weeks of DM, miniature pigs in the DMAB group were transplanted with ABMSCs labeled with superparamagnetic iron oxide (SPIO) (Feridex; Advanced Magnetics, Inc. Cambridge, MA, USA). Miniature pigs in the DMC group were transplanted with the same volume of PBS. The experiment procedure is shown in Fig. 1.

### BMSC isolation, culture and labeling

BMSCs of animals were isolated, cultured and labeled as previously described (31).

Flow cytometry was applied to detect the expression of the following cell markers: CD34-FITC, CD45-FITC, CD29-FITC and CD44-FITC (BD Biosciences) (32).

Third passage BMSCs were labeled with a SPIO nanoparticle contrast agent prior to transplantation. SPIO suspensions with an iron concentration of 50 mg/l were mixed with an equal volume of Poly-L-lysine (PLL) (Sigma-Aldrich) at 1.5 mg/ml for 1 h under gentle agitation. The above ferumoxides-PLL complex was then added to the culture medium and incubated for 48 h. After cells were fixed with 4% ethanol and stained with Prussian blue (PB), light microscopy was performed to assess the iron labeling efficiency. By way of regular fixation, dehydration, embedding, ultrathin section (with the section thickness of 50–80 nm) and staining in turn, cellular ultrastructure and localization of iron in BMSCs were observed under a transmission electron microscope (JEM-1200EX; JEOL Ltd., Tokyo, Japan).

### Transplantation of ABMSCs

Transplantation was carried out after 4 weeks of diabetes in the DMAB group (33). Animals underwent 12-h fasting. Following successful general anesthesia, the animals were fixed to the operating table of digital subtraction angiography (DSA) in the supine position. The right femoral artery was punctured and a micro catheter was catheterized into the dorsal pancreatic artery. The ABMSCs according to their body weight (5×10^5^ cells/kg) were directly injected into the dorsal pancreatic artery (11,34).

### In vivo tracing of ABMSCs following transplantation

The animals were fixed in an experimental special holder and anesthetized as described above. Magnetic resonance imaging (MRI) (3.0T MRI system) was carried out in different sequences 1 week prior to transplantation, and 3 and 6 weeks after transplantation to observe the local signal change of the pancreas (35,36).

### Intravenous glucose tolerance testing (IVGTT)

IVGTTs were carried out prior to establishment of the DM model, transplantation of ABMSCs and 6 weeks after transplantation. Miniature pigs were fasted overnight (12 h), weighed and injected intravenously with a bolus of 50% glucose (0.5 g/kg of body weight) at ear margin venipuncture. Blood was then drawn from an indwelling needle puncture of the external jugular vein at 0, 3, 5, 7, 10, 20, 30, 60 and 120 min after the glucose challenge.

### Oral glucose tolerance testing (OGTT)

At the second day of the IVGTT, miniature pigs were fasted overnight (12 h), weighed and administered glucose (2 g/kg weight) and 25 g mixed feed (dissolved in 100 ml water). Blood was then drawn from an indwelling needle puncture of the external jugular vein at 0, 30, 60, 120 and 180 min after the glucose challenge.

### Measurement of biochemical indices

Blood samples were obtained from an indwelling needle puncture of the external jugular vein and measured for blood glucose (glucose oxidase method). Serum insulin (PI-12K porcine nsulin RIA kit; Millipore Corp., St. Charles, MO, USA) and C-peptide (PCP-22K Porcine C-peptide RIA kit; Millipore Corp.) was quantified by porcine insulin and the C-peptide assay (RIA) kit.

### Histology and immunohistochemistry

Paraffin-embedded pancreatic samples were reviewed histologically using hematoxylin and eosin staining. PB staining was performed to determine intracellular iron using Perls’ reaction method.

Specimens were then deparaffinized with xylene and rehydrated. To block non-specific staining, the sections were incubated in a buffer containing 5% goat serum for 30 min at 37°C. Primary monoclonal antibody of mouse anti-human insulin, glucagon and factor VIII-R (OriGene Technologies, Inc., Beijing, China) were used at dilutions of 1:400, 1:300 and 1:300, respectively, and incubated overnight at 4°C. IgG-conjugated horseradish peroxidase (HRP) and 3,3′-diaminobenzidine tetrahydrochloride (DAB) (Vector Laboratories, Burlingame, CA, USA) were employed to visualize antibody binding.

### Statistical analysis

Statistical data were processed by SPSS 12.0 software. Data with normal distribution were expressed as mean ± SD. One-way analysis of variance (ANOVA) or the unpaired Student’s t-test was performed to determine statistical difference, while the χ^2^ test was used for qualitative data. Two-way analysis of variance (ANOVA) was used to determine the effects of treatement and period of time, followed by Bonferroni group comparisons. P<0.05 was considered to indicate statistical significance.

## Results

### Hyperglycemia was controlled and insulin use was reduced following transplantation of ABMSCs

**Figure 2:**
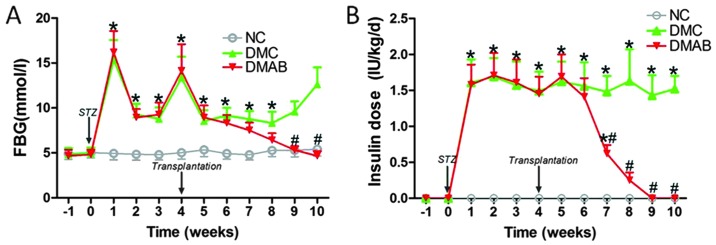
Marked changes of fasting blood glucose (FBG) and insulin dosage over time in different groups. Time-course panels had significant effects of treatment and time by two-way analysis of variance (ANOVA) followed by Bonferroni group comparisons. For all panels: ^*^P<0.05 vs. normal controls (NC) group; ^#^P<0.05 vs. diabetic minipig control group (DMC).

No significant difference was observed in blood glucose during 1–4 weeks after establishment of the type 1 diabetes model between the DMC and DMAB groups (Fig. 1A). However, the FBG level was gradually reduced, and maintained between 2.3 and 7.5 mmol/l after ABMSC transplantation (Fig. 2A). Moreover, the insulin dose was gradually decreased and discontinued during the 17–28th day post-operation (Fig. 2B).

### β-cell function was partially restored in the DMAB group

**Figure 3:**
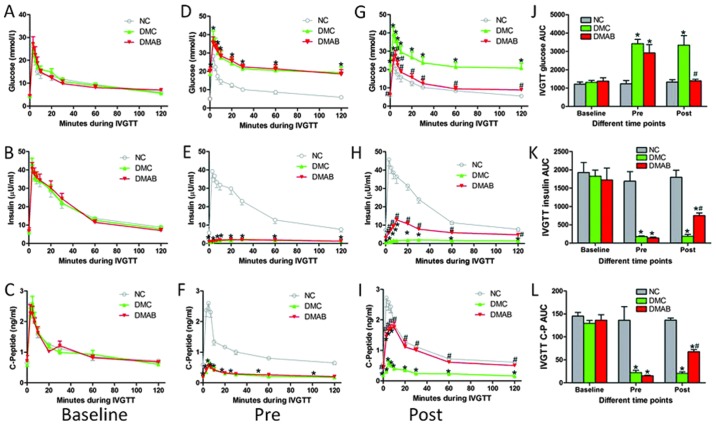
Glucose, insulin and C-peptide responses during intravenous glucose tolerance testing (IVGTT) among the three groups at different time points. (A–C) Blood glucose, insulin and C-peptide levels during IVGTT showed no significance among the three groups at baseline, respectively. (D–F) Blood glucose, insulin and C-peptide levels during IVGTT were measured among three groups at (D–F) pre-autologous BMSCs (ABMSCs), respectively and (G–I) post-ABMSCs, respectively. (J–L) Glucose, serum insulin and C-peptide area under the curve (AUC) during IVGTT at different time-points among the three groups were analyzed. Time-course panels had significant effects of treatment and time by two-way analysis of variance (ANOVA) followed by the Bonferroni group comparisons. For all panels: ^*^P<0.05 vs. normal controls (NC) group; ^#^P<0.05 vs. diabetic minipig control group (DMC).

IVGTT showed that there was no significance among the three groups in terms of blood glucose, serum insulin and C-peptide level at the baseline (Fig. 3A–C). The blood glucose, serum insulin and C-peptide levels in the DMC and DMAB groups at the time point of pre-ABMSCs were in line with the characteristics of T1D (Fig. 3D–F). Insulin and C-peptide curves showed a flat form. At the end-point of our experiment, there was no obvious change of blood glucose, serum insulin and C-peptide level in the NC and DMC groups compared to those prior to ABMSC transplantation (Fig. 3G–I). The peak of blood glucose in the DMAB group was significantly reduced, and the curve was close to the normal glucose tolerance (NGT) curve (Fig. 3G). Insulin and C-peptide secretion levels prior to and after glucose load were significantly higher than those of DMC group, which presented a small secretion peak between 3 and 10 min, and was gradually reduced to basic level (Fig. 3H and I).

**Figure 4:**
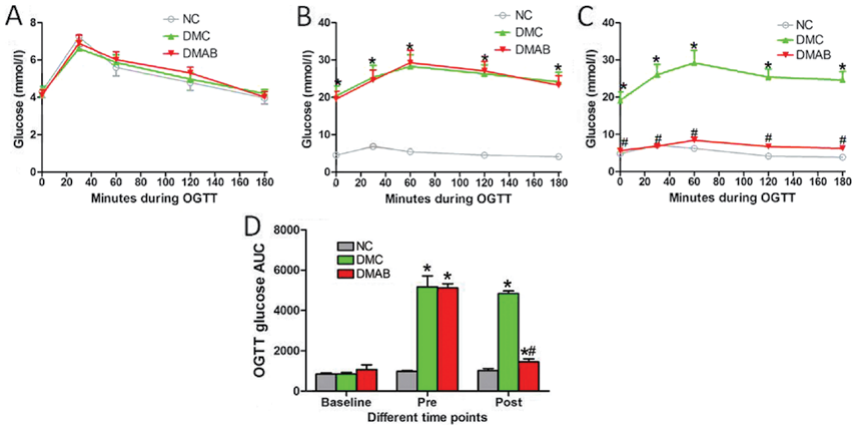
Glucose responses during oral glucose tolerance testing (OGTT) among the three groups at (A) baseline, (B) pre-autologous BMSCs (ABMSCs) and (C) post-ABMSCs. (D) Respective area under the curve (AUC) values during OGTT. ^*^P<0.05 vs. normal controls (NC) group, ^#^P<0.05 vs. diabetic minipig control group (DMC).

For the area under the curve (AUC) of blood glucose, serum insulin and C-peptide level in IVGTT, there was no significance among the three groups at the baseline (Fig. 3J–L). Prior to ABMSC transplantation, AUC of blood glucose in the DMC and DMAB groups was significantly greater than that of the NC group (Fig. 3J), whereas AUC of serum insulin and C-peptide in the DMC and DMAB groups was significantly less than those of the NC group, respectively (Fig. 3K and L). There was no significant difference of AUC between the DMC and DMAB groups (Fig. 3K and L). At the end-point of our experiment, AUC of blood glucose in the DMAB group was less than that prior to ABMSC transplantation, although it was not significantly different compared to that of the baseline level (Fig. 3J). The AUCs of insulin and C-peptide in the DMAB group were significantly more than those of the DMC group (Fig. 3K and L). OGTT showed a similar trend to IVGTT for the three groups (Fig. 4A–D).

### PB staining was positive in pancreas with transplanted ABMSCs

**Figure 5:**
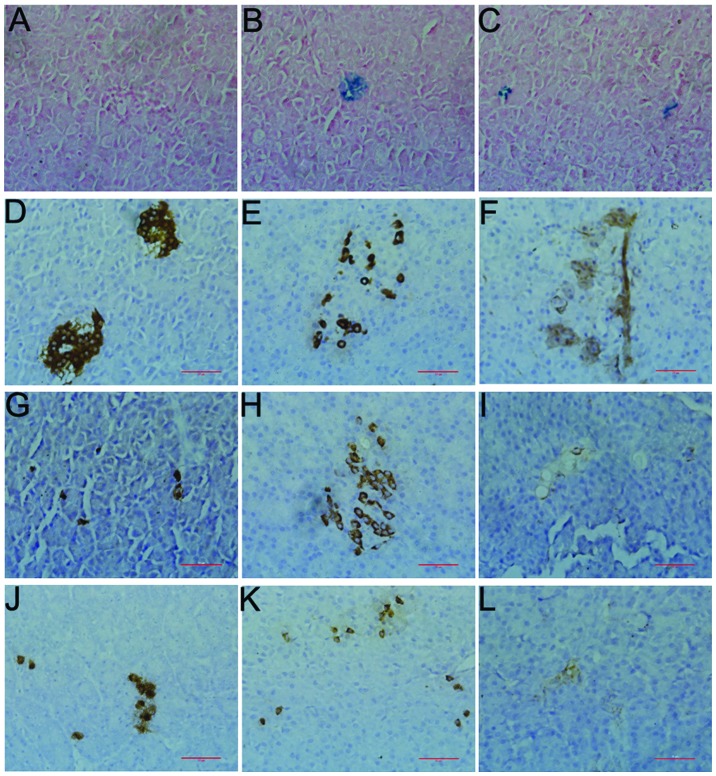
Representative histology of pancreatic sections with Prussian blue (PB) staining and immunohistochemistry (magnification, ×40). (A) There was no positive staining in the normal control (NC) group; there was positive PB staining in the (B) islet cells and (C) pancreatic ductal epithelial cells in diabetic minipigs with autologous BMSCs transplantation group (DMAB). Representative immunohistochemistry of pancreatic sections stained with (D) anti-insulin antibody, (E) anti-glucagon antibody and (F) anti-factor-VIII antibody in normal minipigs [normal controls (NC)]; representative immunohistochemistry of pancreatic sections stained with (G) anti-insulin antibody, (H) anti-glucagon antibody and (I) anti-factor-VIII antibody in streptozotocin (STZ)-diabetic minipigs [Diabetic minipig control group (DMC)]; representative immunohistochemistry of pancreatic sections stained with (J) anti-insulin antibody, (K) anti-glucagon antibody and (L) anti-factor-VIII antibody in STZ-diabetic minipigs transplanted with ABMSCs (DMAB).

The positive PB staining cells in the pancreas of miniature pigs showed that the islet and pancreatic ductal epithelial cells derived from the SPIO-labeled BMSCs. The cells were positive in the DMAB group (Fig. 5B–C) but not in the NC group (Fig. 5A), suggesting that SPIO-labeled BMSCs were implanted in the islet following transplantation, differentiated into islet cell or formed new islet, or implanted in exocrine pancreas and differentiated into pancreatic ductal epithelial cells.

### Small islets were regenerated in pancreas after transplantation of ABMSCs

As shown in Fig. 5D–F, there was an abundance of islets; the boundary was clear; the cell mass shape was circular, oval, or irregular, and present in alignment, with plump cytoplasm, and a circular nucleus in the NC groups. Capillary was rich around the islet or penetrating the internal islet. By contrast, the islets were scarce, the boundary was not clear, and the contour was incomplete or collapsed in the DMC groups. Additionally, the cells were arranged in a disorderly manner, the nuclear size and shape were irregular, and some of the islet cells appeared to have vacuolar degeneration, i.e., few β cells remained while the α cells became the main pancreatic islet cells. Capillaries were significantly reduced and unevenly distributed (Fig. 5G–I).

New small islets were identified in the majority of islets that were distributed in different parts of the pancreas in the DMAB groups. The new islets and survival islets coexisted. The size and number of the new islets were obviously fewer than those of the NC groups. The contour of the cells was regular, and cells were distributed uniformly, with the cytoplasm being much richer and light dyed lighter compared with the NC groups. The nuclear size and form were normal. There were more β cells present as compared with α cells, and capillaries were more abundant than those of the DMC group (Fig. 5J–L).

### Successful real-time tracing of transplanted ABMSCs by MRI

**Figure 6:**
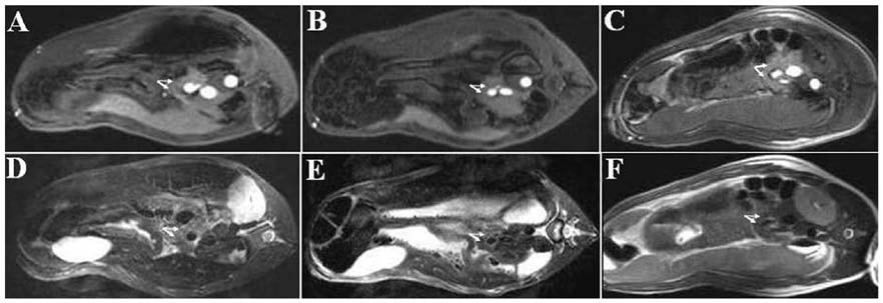
Magnetic resonance imaging (MRI) of diabetic minipigs with autologous BMSCs transplantation group (DMAB) pancreas of minipigs at three time-points in different sequences. (A) TFL T_1_WI MRI at 1 week pre-autologous BMSCs (ABMSCs), (B) TFL T_1_WI MRI at 3 weeks post-ABMSCs, (C) TFL T_1_WI MRI at 6 weeks post-ABMSCs, (D) TSE T_2_WI MRI at 1 week pre-ABMSCs, (E) TSE T_2_WI MRI at 3 weeks post-ABMSCs and (F) TSE T_2_WI MRI at 6 weeks post-ABMSCs. Pancreases are shown (arrows).

At 1 week before transplantation, 3 and 6 weeks after transplantation, there was no significant change among MRI TFL T_1_WI signals of the pancreases in the DMAB group (Fig. 6A–C). However, the sporadic mottled low-signal area emerged distinctly in the pancreases of DMAB animals in MRI TFL T_2_WI at 1 week prior to transplantation, and 3 and 6 weeks after ABMSC transplantation (Fig. 6D–F).

### PB staining of BMSCs

**Figure 7:**
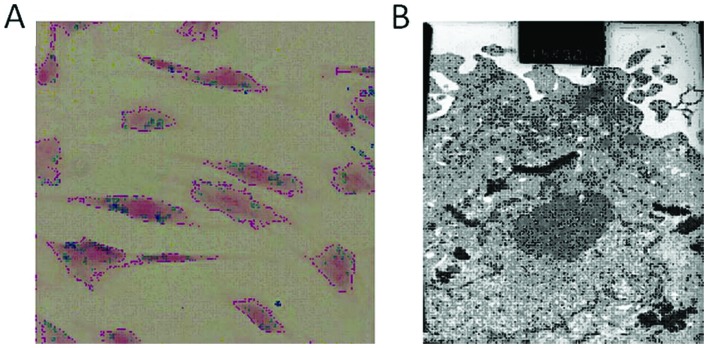
Assessment of Prussian blue (PB) staining bone marrow-derived mesenchymal stem cells (BMSCs). (A) Superparamagnetic iron oxide (SPIO)-labeled BMSCs under light microscopy (PB staining; magnification, ×200) and (B) transmission electron microscopy (×11,500).

SPIO-labeled BMSCs showed numerous blue particles in the cytoplasm of the labeled cells and nano-iron particles near the nucleus (Fig. 7A and B).

## Discussion

T1D is obtained from the specific autoimmune reaction of β cells induced by a variety of pathogenic factors, with 20–30% of viable β cells prior to onset of typical clinical symptoms. However, autoimmune reaction begins to accelerate after typical clinical manifestations, similar to β-cell damage (12). Thus protection of the remaining β cells and promotion of intrinsic β-cell regeneration and new islet cell formation has become the focus of study in T1D.

### Islet function is restored following ABMSC transplantation

A deep vein path was established in order for OGTT and IVGTT to be evaluated repeatedly (13–15). Three different time-points were selected, including the baseline before T1D model, and time-points pre- and post-ABMSCs. The results showed that the OGTT and IVGTT glucose curve was significantly lower than that in the DMC group, albeit similar to that of the NC group at the end-point of the experiments. Moreover, there was improvement in the basal and post-glucose-challenge insulin and C-peptide secretion in the DMAB group, with the insulin and C-peptide secretion peaks evident 3–10 min after intravenous injection of 50% glucose, which suggests that the initial phase insulin release of β cells in the DMAB group was significantly improved. In addition, the exogenous insulin use gradually decreased after operation until the discontinuation of insulin in the DMAB group, with the FBG being maintained at normal and relatively stable levels. The results of this study indicate that ABMSCs can effectively restore the islet, improve β-cell function and glucose tolerance, and reverse the hyperglycemic conditions in early STZ-induced T1D.

Results of previous animal studies have shown that peripheral vein infusion or pancreatic local injection of autologous HSC or BMSCs can improve the islet function of mice (16,17), and miniature pigs (18), and reverse hyperglycemic conditions 15–30 days after transplantation. A follow-up clinical trial lasting almost 5 years (average 29.8 months) showed that peripheral venous transplantation of autologous HSC joint with immune intervention therapy can improve the islet function of new developed T1D patients; 20 cases (23 cases in total) stopped exogenous insulin treatment, and 12 patients had discontinued insulin for ≥31 months (19,20). The abovementioned data suggest that stem cell transplantation is expected to become an effective means for the early treatment of T1D. However, the abovementioned studies have certain limitations such as non-autologous cell transplantation, lack of different time window of cell transplantation, non-directional pancreatic transplantation, more severe trauma, or lack of standardization and quantitation in grafts (21,22). In the present study, we transplanted autologous and quantitative BMSCs into pancreas by super microcatheter targeting into pancreas dorsal artery in the early stage T1D pigs, which has solved problems including time window selection, optimization of transplantation procedure, graft standardization and quantification in the treatment of T1D with autologous stem cell transplantation.

### In vivo tracing of transplanted autologous cells

SPIO is a negative contrast agent used in magnetic resonance and has been approved for clinical diagnosis by FDA. It is identified by the reticuloendothelial system after intravenous injection, and is absorbed by phagocytes. The corresponding regional signal is reduced following phagocytosis of SPIO particles. In this study, we have established a safe, real-time, dynamic, and non-invasive MRI tracing technique in T1D miniature pigs. We observed uneven pancreas signal in T_2_WI at post-operative 3 and 6 weeks, an obvious scattered low-signal area, and local low-signal pancreas presented s gradually decreasing trend with the passage of time. Thus MRI may be used for short-term tracing and observing the distribution and survival of SPIO-labeled ABMSCs in the pancreas of miniature pigs.

SPIO-labeled islet is clearly observed by MRI imaging 18 weeks after transplantation (23). The 1.5T MRI system shows the body distribution of SPIO-labeled BMSCs transplanted into brain (24), and heart (25) in rats, mice and miniature pigs. The real-time tracing time of SPIO-labeled BMSCs is relatively shorter compared to that of real-time islet transplantation tracing; however, it may still be used for short-term tracing in the clinic.

### The role of ABMSCs in islet repairing and remodeling

Immunohistochemistry of pancreas showed that PB staining of some islet and pancreatic ductal epithelial cells was positive, while insulin, glucagon and vascular endothelial VIII factor staining were visible. The islets were mainly new small islets in the DMAB group, which coexisted with survival islets. The area of new islets and cell number were significantly less than those of normal islets. The majority of cells in the new islets were β cells, with α cells a minority. This result suggests that directional transplanted ABMSCs may be implanted in the pancreas and differentiated into islet and pancreatic ductal epithelial cells in miniature pigs. However, whether ABMSCs restore islets hyperplasia by promoting pancreatic inherent stem cell differentiation into islet cells, self-replicate into inherent islet cells in a paracrine manner should be investigated.

BMSCs are capable of repairing and remodeling the islets by promoting intrinsic islet cell replication (26) and the formation of new islets in type 1 diabetic mice (10,27). Transplanted BMSCs differentiate into endothelial cells, improve local microcirculation (28) and promote the repair of diabetic neuropathy by secreting vascular endothelial growth factor (VEGF) and basic fibroblast growth factor (bFGF) in in a paracrine manner (28,29).

In summary, quantitative ABMSC transplantation targeted into the pancreas can effectively improve the islet function of miniature pigs with early T1D, reverse high blood glucose state, discontinue exogenous insulin therapy in the short term and maintain normal blood glucose. MRI scanning can be used for dynamic, non-invasive and real-time tracing of SPIO-labeled ABMSCs in the short term after transplantation.
